# The high-quality *Pinellia pedatisecta* genome reveals a key role of tandem duplication in the expansion of its agglutinin genes

**DOI:** 10.1093/hr/uhac289

**Published:** 2022-12-30

**Authors:** Zhihao Qian, Jun Ding, Zhizhong Li, Jinming Chen

**Affiliations:** Wuhan Botanical Garden, Chinese Academy of Sciences, Wuhan 430074, China; University of Chinese Academy of Sciences, Beijing 100049, China; CAS Key Laboratory of Plant Germplasm Enhancement and Specialty Agriculture, Wuhan Botanical Garden, Innovative Academy of Seed Design, Chinese Academy of Sciences, Wuhan, China; Wuhan Botanical Garden, Chinese Academy of Sciences, Wuhan 430074, China; Wuhan Botanical Garden, Chinese Academy of Sciences, Wuhan 430074, China

Dear Editor,

*Pinellia* Tenore*,* a small genus of the monocot family Araceae, consists of only nine perennial herbaceous species and is mainly distributed in East Asia [1]. *Pinellia* plants have been widely used as herbal medicines in Asia for over 2000 years. Among these species, *P. ternata* and *P. pedatisecta* are most widely used as traditional medicinal herbs [2]. In China, the medicinal utilization of *P. ternata* and *P. pedatisecta* was first documented in the Divine Farmer’s Materia Medica (Chinese name: ‘Shennong Bencao Jing’) during the Eastern Han dynasty (25–250 AD). Tubers produced by these plants have been traditionally utilized to treat vomiting, infection, and inflammation [3]. Modern pharmacological studies have indicated that the pharmacological effects of *Pinellia* plants are closely related to endogenous components, such as plant lectins, alkaloids, amino acids, nucleosides, and polysaccharides [2]. *P. ternata* has been listed in the Chinese Pharmacopoeia as a common traditional Chinese medicine. However, to date no genomic resources have been reported in the genus *Pinellia*, which greatly limits further studies on this valuable resource.

Here we report a high-quality *P. pedatisecta* genome using Illumina, PacBio, and Hi-C sequencing technologies. The assembled genome was 1182.37 Mb in size, consistent with the estimated results of flow cytometry and *k*-mer analysis, comprising 13 pseudochromosomes with a contig N50 and scaffold N50 length of 17.26 and 85.81 Mb, respectively (Fig. 1A, Supplementary Data Figs S1–S3). A total of 77.66% repetitive regions were identified in the *P. pedatisecta* genome. Approximately 39.23% of the repetitive elements were long terminal repeat retrotransposons (LTR-RTs), including Gypsy (24.67%) and Copia (14.53%;Supplementary Data Table S1). Also, we observed that both Gypsy and Copia experienced two obvious bursts at ~0.4 and ~2 million years ago (Mya), respectively (Supplementary Data Fig. S4). Moreover, 26 113 protein-coding genes were predicted in the genome, of which 92.39% were functionally annotated among six public databases (Supplementary Data Table S2). Additionally, Benchmarking Universal Single-Copy Orthologs (BUSCO) assessment revealed there were 91.3 and 96.2% complete BUSCOs in *P. pedatisecta* genome and gene sets, respectively (Supplementary Data Table S3). The QV score of the *P. pedatisecta* genome is 33.37, corresponding to 99.38% accuracy, indicating that the *P. pedatisecta* genome was nearly complete and highly accurate (Fig. 1A).

**Figure 1:**
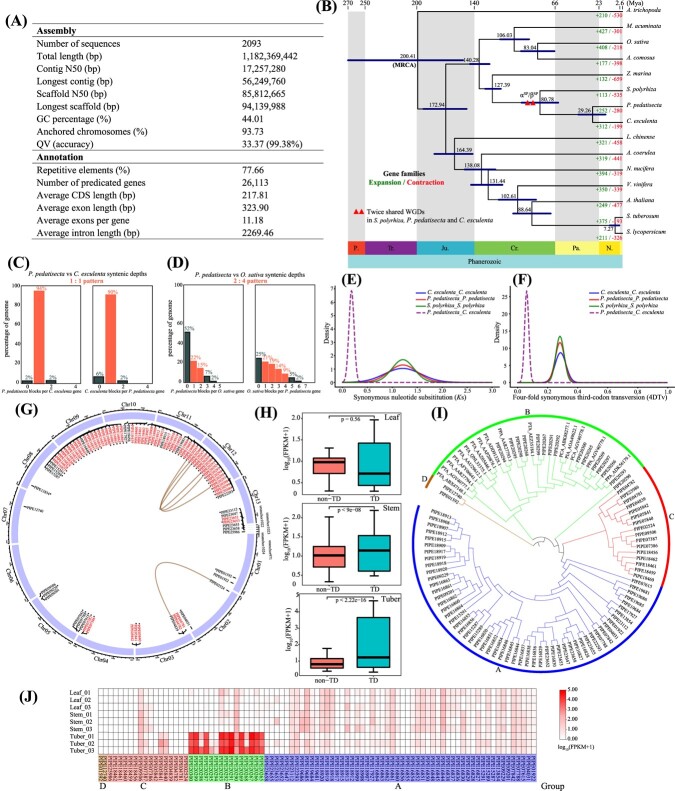
The *P. pedatisecta* genome. (A) Statistics of *P. pedatisecta* genome assembly. (B) Phylogenetic tree showing divergence times and the evolution of gene families in *P. pedatisecta*. Estimated divergence times (Mya) are shown at each node. Expansion and contraction of gene families are denoted as numbers in green and red, respectively. The red triangles denote the two WGDs reported in Wang *et al*. [5]. (C) Syntenic depths between *P. pedatisecta* and *C. esculenta*. (D) Syntenic depths between *P. pedatisecta* and *O. sativa*. (E) *K*_s_ distribution of syntenic genes from *S. polyrhiza*, *P. pedatisecta*, and *C. esculenta*. (F) 4DTv density profiles of paralogs and orthologs for *S. polyrhiza*, *P. pedatisecta*, and *C. esculenta*. (G) Chromosomal distribution of 87 PPA genes. Red font represents tandem-duplicated PPA genes. Asterisks indicate that the gene is supported by transcriptome evidence. PPA genes generated by WGDs are linked by lines. (H) Expression differences of tandem-duplicated PPA genes and non-tandem-replicated PPA genes in three tissues (*P*-value is from the Wilcoxon test). (I) Phylogenetic tree of PPA genes from 16 *Pinellia* lectins downloaded from NCBI and 87 PPA genes. (J) Heat map showing three tissues with *P. pedatisecta*-specific expressions of PPA genes.

The phylogenetic tree constructed for *P. pedatisecta* and 14 other representative plants using 485 common single-copy ortholog genes showed that *P. pedatisecta* diverged from the close relative species *Colocasia esculenta* ~29.26 Mya (Fig. 1B). Furthermore, gene family clustering analysis revealed that 252 and 280 gene families showed significant expansion and contraction in the *P. pedatisecta* genome, respectively. GO and KEGG enrichment analyses showed that 280 significantly expanded gene families were enriched for the GO terms ‘secondary metabolic process’, ‘cellular amide metabolic process’, and ‘amide biosynthetic process’, as well as the KEGG pathways ‘isoflavonoid biosynthesis’, ‘phenylpropanoid biosynthesis’, and ‘metabolic pathways’, which likely associate with the synthesis and accumulation of secondary metabolites (Supplementary Data Fig. S5).

Whole-genome duplication (WGD) events have been inferred to play important roles in plant genome evolution and function [4]. Our syntenic analysis showed that the syntenic depth between *P. pedatisecta* and *C. esculenta* and between *P. pedatisecta* and *Spirodela polyrhiza* (two WGDs) was 1:1, and that between *P. pedatisecta* and *Oryza sativa* (three WGDs) was 2:4, indicating that there were two rounds of WGDs in *P. pedatisecta* (Fig. 1C and D, Supplementary Data Fig. S6A). Syntenic dotplots also provided clearly visual evidence for two WGDs in *P. pedatisecta* (Supplementary Data Fig. S6B–E). Previous studies have shown that two WGDs (α^SP^/β^SP^) occurred within a short period at 95 Mya in *S. polyrhiza*, and were shared between *C. esculenta* and *S. polyrhiza* [5, 6]. In this study, *S. polyrhiza* diverged to the most recent common ancestor of *P. pedatisecta* and *C. esculenta* at ~80.78 Mya. Also, the *K*_s_ and 4DTv (four-fold synonymous third-codon transversion) distributions showed a single peak at the identical position for *P. pedatisecta*, *S. polyrhiza*, and *C. esculenta*, indicating that the two WGDs occurring within a short period were shared in these three species (Fig. 1B, E, and F). In addition, GO and KEGG enrichment analyses were performed to determine the functional roles of the 3448 genes retained after WGD events. These genes retained after WGDs were significantly enriched in ‘regulation of the biosynthetic process’, ‘heterocycle biosynthetic process’, ‘nucleobase-containing compound biosynthetic process’, and ‘aromatic compound biosynthetic process’ (Supplementary Data Fig. S7), suggesting that WGDs likely contribute to the diversification of metabolites and the accumulation of medicinal activities in the medicinal plant *P. pedatisecta*.

*Pinellia pedatisecta* agglutinin (PPA), belonging to the *Galanthus nivalis* agglutinin (GNA) family, is a specific mannose-binding plant lectin, as well as an important medicinal component of *P. pedatisecta* with physiological effects such as bacteriostatic, insecticidal, and antitumor activities [2,7]. In our study, 87 PPA genes were identified across the whole genome using PF01453 as a query. Chromosomal localization showed an uneven distribution of PPA genes, with the most distributed on chromosome 10, with 37 PPA genes. In addition, 68.97% (60) and 11.49% (10) of the PPA genes were classified to have undergone tandem duplication (TD) and WGD events based on intraspecific syntenic analysis, respectively, and eight genes were replicated in both TD and WGD events (Fig. 1G). These results suggested that the formation and expansion of the PPA genes in *P. pedatisecta* were mainly driven by TD events to generate new gene copies in tightly linked genomic clusters. A similar evolutionary pattern was also found in the study of *Cucumis sativus* lectins [8]. Furthermore, 45 out of 60 tandem-duplicated PPA genes were supported by transcripts (Fig. 1G). Notably, the expression of tandem-duplicated PPA genes was significantly higher than that of non-tandem-duplicated PPA genes in stems and tubers (Fig. 1H), indicating that gene duplication greatly affects gene dosage and generally leads to high gene expression [9]. The *K*_a_/*K*_s_ ratios of both TD and WGD gene pairs were estimated to be <1 (Supplementary Data Table S4), revealing that most PPA genes underwent purifying selection to maintain their functionality.

To obtain a more comprehensive insight into the GNA gene family in *P. pedatisecta*, we performed a phylogenetic analysis of *P. pedatisecta* and four closely related species in Araceae (Supplementary Data Fig. S8). All identified GNA genes of five species were clustered into four major groups (A–D). We detected that the GNA genes in *P. pedatisecta* underwent lineage-specific expansions, especially in group B, which was likely to be associated with recent TD events. To understand the evolutionary relationships among the GNA genes in *Pinellia*, a phylogenetic tree was constructed by combing 16 GNA genes from four *Pinellia* species retrieved from NCBI with 87 PPA genes newly identified here (Fig. 1I). Conserved motif analysis detected 10 conserved protein motifs, and motif 2, as part of the B_lectin domain, was present in all PPA genes. Meanwhile, genes within the same phylogenetic group presented similar conserved structural characteristics (Supplementary Data Fig. S9). In addition, all 16 retrieved GNA genes and 15 PPA genes newly identified here were clustered into group B (Fig. 1I). In previous studies, some of PPA genes in group B were proven to enhance resistance to aphids in transgenic plants and inhibit cancer cell proliferation [10,11]. Notably, all PPA genes from *P. pedatisecta* in group B (except *PIPE20294*) included two B_lectin structural domains, which likely associated with aphid resistance and anticancer. Additionally, all PPA genes in group B were relatively highly expressed in tubers (Fig. 1J), which can explain why the dried tubers of *P. pedatisecta* are the main tissues used in folk medicine.

In summary, the high-quality assembly of the *P*. *pedatisecta* genome provides a forceful reference for studying *Pinellia* spp. herbs. Also, the identification of *P*. *pedatisecta* agglutinin genes in this study will give a large potential for novel investigations and practical applications in biomedicine and agriculture.

## Acknowledgements

This work was supported by grants from the Strategic Priority Research Program of Chinese Academy of Sciences (No. XDB31000000).

## Author details

J.M.C. and Z.Z.L. designed the study and led the research. Z.H.Q. wrote the draft manuscript and analyzed the data. J.M.C., J.D., and Z.Z.L. contributed substantially to the revisions. The final manuscript has been read and approved by all authors.

## Data availability

All data sets (Illumina, PacBio, Hi-C, RNA-seq and the genome assembly) have been deposited at the China National GeneBank DataBase (CNGBdb, https://db.cngb.org/) website under the accessions CNS0561814–CNS0561818 with CNGB-Project ID CNP0003127.

## Conflict of interest

The authors declare that they have no conflict of interest.

## Supplementary data

Supplementary data is available at *Horticulture Research Journal* online.

## Supplementary Material

Web_Material_uhac289
