# Supplementary material for: The high-quality Pinellia pedatisecta genome reveals a key role of tandem duplication in the expansion of its agglutinin genes
Source: Hortic Res. 2022 Dec 30;10(3):uhac289. doi: 10.1093/hr/uhac289 (PMC10015338; doi:10.1093/hr/uhac289)
Supplement: Web_Material_uhac289 [file web_material_uhac289.docx]

**Supplemental Information for:**

**The high-quality** ***Pinellia pedatisecta* genome reveals a key role of tandem duplication in the expansion of its agglutinin genes**

Zhihao Qian^1,2^, Jun Ding^3^, Zhizhong Li^1^*, Jinming Chen^1^*

*^1^ Wuhan Botanical Garden, Chinese Academy of Sciences, Wuhan 430074, China*

*^2^ University of Chinese Academy of Sciences, Beijing 100049, China*

*^3^ CAS Key Laboratory of Plant Germplasm Enhancement and Specialty Agriculture,*

*Wuhan Botanical Garden, Innovative Academy of Seed Design, Chinese Academy of*

*Sciences, Wuhan, China*

**Running head: A high-quality *Pinellia pedatisecta* genome**

*Authors for correspondence:

**Zhizhong Li**, E-mail: lizhizhong@wbgcas.cn

**Jinming Chen**, E-mail: jmchen@wbgcas.cn

**1. Supplementary note**

**1.1. Sampling and genome size estimation**

The sequenced sample of *Pinellia pedatisecta* (2n = 26) was collected at the Wuhan Botanical Garden, Chinese Academy of Sciences (30° 33′ N, 114° 24′ E) in Hubei Province, China. High-quality DNA was extracted from the fresh young leaves of the plant using the MagicMag Plant Genomic DNA Micro Kit (Sangon Biotech Co., Shanghai, China).

First, the *P. pedatisecta* genome size was estimated using flow cytometry with a BD AccuriTMC6 flow cytometer (BD Biosciences, San Jose, CA, USA) and the *Nelumbo nucifera* genome (genome size = 807.6 Mb) was utilized as a reference [1]. The *k-mer* analysis was conducted as the second strategy to evaluate the genome size with Illumina paired-end short reads using Jellyfish v2.1.3 [2]. The heterozygosity and repeat content were estimated using GCE v1.0.0 (https://github.com/fanagislab/GCE) based on *k-mer* (*k* = 17) analysis.

Then, the size of the *P. pedatisecta* genome, estimated using flow cytometry, was approximately 1036 Mb (Figure S1). The *k-mer* analysis further showed that the estimated genome size was 1177 Mb with low heterozygosity (0.25%) and high repeat content (62.48%; Figure S2; Table S5).

**1.2. Genome sequencing and *de novo* genome assembly**

Three sequencing methods (Illumina, PacBio, and Hi-C sequencing) were employed to sequence the genome in this study. The Illumina and PacBio libraries were constructed and sequenced by following the standard protocol described by Qian et al. [3]. Then, 108.81 Gb (approximately 92× sequence depth) Illumina paired-end reads and 136.23 Gb (about 115× sequence depth) PacBio long reads were obtained (Table S6). The Hi-C libraries were sequenced on the Illumina HiSeq X Ten platform (Illumina, San Diego, CA, USA) with the 150PE mode following a standard procedure described previously [4].

The *P. pedatisecta* genome was *de novo* assembled based on PacBio long reads using Canu v1.8 [5] with [default](javascript:;) parameters, except for the corrected ErrorRate set to 0.035. The preliminary assembled genomes were polished using Illumina short reads with Pilon v1.23 in three iterative rounds [6]. The chromosome-scale assembly was performed using Hi-C scaffolding. Approximately 7.8 × 10^8^ Hi-C clean read pairs were obtained for anchored contigs after the quality control was mapped to the draft genome using BWA v0.7.17 in default settings [7]. Lachesis [8] was used to remove the sequences beyond 500 bp from the restriction site, and it was further conducted for clustering, ordering, and orienting the mapped data. Finally, JuiceBox v1.8.8 [9] was applied to visualize the Hi-C map and manually correct the assembly errors. Benchmarking Universal Single-Copy Orthologs (BUSCO v4.0.6) [10] was applied to assess the integrity of the genome assembly using the embryophyta_odb10 database. In addition, QV and completeness scores were estimated using Merqury v1.3 [11] (parameters *k*=20 and tolerable collision rate: 0.001) based on Illumina short-reads.

**1.3. RNA extraction and transcriptome sequencing**

Seven tissues from *P. pedatisecta*, including the leaf, stem, bract, pistil, [stamen](javascript:;), [fruit,](javascript:;) and tuber, were collected for transcriptome sequencing. The total RNA of each tissue was isolated using the plant RNA isolation kit (DP432; Tiangen Technologies, Beijing, China). The RNA-seq libraries were constructed using the Truseq Stranded RNA Library Prep Kit (Illumina), followed by sequencing on the NovaSeq 6000 platform.

**1.4. Repeat element identification and** **genome annotation**

The *de novo* repeat library of the *P. pedatisecta* assembly was constructed using RepeatModeler v2.0.1 [12] with default parameters. Moreover, RepeatMasker v4.0.7 (http://www.repeatmasker.org) was used to identify the repeat elements using the *de novo* repeat library and the default library from the RepeatMasker database (http://www.Repeatmasker.org). LTR_FINDER v1.07 [13] and LTRharvest [14] were employed to identify the long terminal repeat-retrotransposons (LTR-RTs) using default parameters. LTR_retriever v2.9.0 [15] was used for the accurate identification of the LTR-RTs using the integrative results of LTR_FINDER and LTRharvest. The LTR insertion time was estimated using the LTR_retriever with the substitution rate of *Oryza sativa* (1.3e-8 per bp per year) [16].

Coding gene prediction analysis in the *P. pedatisecta* genome was conducted using a combination of *ab initio* and homology- and transcriptome-based pipelines with the repeat-masked genome. The *ab initio* annotation was performed using Braker2 v2.1.2 [17] with default parameters. The homology-based annotation was performed using GeMoMa v1.7.1 [18] with four closed species of *Zostera marina*, *Lemna minor*, *Spirodela polyrhiza*, and *Colocasia esculenta*. The transcriptome-based annotation was performed using the following two strategies: 1) the RNA-seq data were mapped to the *P. pedatisecta*genome using HISAT2 v2.2.1 [19], and then the StringTie v1.3.3 [20] was conducted to improve the transcriptome structure predictions; 2) Trinity v2.8.5 [21] was applied to assemble the RNA-seq data, followed by the prediction of the gene structures using PASA v2.4.1 [22]. Finally, all results of all these predictions were integrated using EVM v1.1.1 [23], and PASA was used to update the results of EVM.

Functional annotation of the protein-coding genes was performed using BLASTP v2.2.31 (E-value = 1E-05) against six protein databases, namely the GO [24], KEGG [25], KOG (https://ftp.ncbi.nih.gov/pub/COG/KOG/), NR (https://ftp.ncbi.nlm.nih.gov/), InterPro [26], and Swiss-Prot (<http://www.expasy.ch/sprot>) databases.

**1.5. Phylogenetic and gene family analyses**

The protein sequences of *P. pedatisecta* and 14 other representative plants (Table S7) were selected for gene family clustering using Orthofinder v2.5.2 [27]. We used MUSCLE v3.8 [28] to align the protein sequences of single-copy orthologous genes and convert the alignments into codon sequences by PAL2NAL v14.1 [29]. After Gblocks v0.91b [30] was employed to filter the non-conservative regions, we extracted the 1st and 2nd codon positions from aligned codon sequences to construct the phylogenetic trees using IQ-TREE v2.1.2 [31]. The divergence time was estimated using the MCMCtree program in the PAML v4.9 (http://abacus.gene.ucl.ac.uk/software/paml.html). The divergence times of the crown group of monocots (184–131 million years ago, Mya) [32], the crown group of eudicots (161–125 Mya) [32], and *Z. marina*–*S. polyrhiza* (134–117.7 Mya) from the TimeTree database (<http://www.timetree.org/>) were utilized for calibration. We used CAFE 5 [33] to detect the gene family size changes in each species and then determined the expansion and contraction of the gene families at each branch with *P*-value < 0.05.

**1.6. Whole-genome duplication analysis**

The paralogous genes between *P. pedatisecta*, *S. polyrhiza*, *C. esculenta*, and *O. sativa* were identified using BLASTP (E-value = 1E-05). McScanX [34] was used to identify the syntenic blocks using default parameters. The synonymous substitution rate (*Ks*) values of the syntenic blocks were calculated using the YN00 program in the PAML package [35]. The whole-genome duplication (WGD) events were estimated based on the four-fold synonymous third-codon transversion (4DTv) and *Ks* distributions. Then, according to the method of Wang et al. [36], we performed evolutionary rate correction by aligning the *Ks* and 4DTv peaks of *S. polyrhiza* to the corresponding location in the *P. pedatisecta* *Ks* and 4DTv distribution. The density distribution map of the 4DTv and Ks values was illustrated using WGDI [37].

**1.7. The identification and characterization of *P. pedatisecta* agglutinin genes**

In order to identify the *Galanthus nivalis* agglutinin (GNA) genes in *P. pedatisecta*, *S. polyrhiza*, *C. esculenta*, *Pistia stratiotes*, and *Amorphophallus konjac* (Table S7), we used PF01453 as a query from Pfam database (http://pfam.xfam.org) to search for GNA genes in five species genomes by the software HMMER v3.2 (E-value 1e-05) [38]. To remove potentially false-positive candidate GNA genes, the online NCBI Conserved Domain Database (CDD) and InterProScan v5.51 [39] were used to verify the protein domains of all identified candidate genes. The tandem duplication and WGD events of *P. pedatisecta* agglutinin gene **(**PPA) were determined using MCScanX. KaKs_Calculator v2.0 [40] was used to calculate non-synonymous substitutions (*Ka*) and synonymous substitutions (*Ks*) of paralogous PPA genes.

The 16 *Pinellia* lectin genes were retrieved from NCBI (https://www.ncbi.nlm.nih.gov/) (Table S8). Then, 16 *Pinellia* lectin genes and 87 PPA genes, and GNA genes of five species were multiple alignments by MUSCLE version 3.8., respectively. The NJ tree with 1000 bootstraps was constructed using protein alignments based on the JTT model in MEGA7 [41]. TBtools v1.098769 [42] was used to visualize the conserved domain of PPA genes identified by MEME v5.5.0 (http://meme-suite.org/tools/meme, accessed on 10 October 2022) and to display the physical location of PPA genes in the *P. pedatisecta* genome. To investigate the expression patterns of the PPA genes in different tissues, leaves, stems, and tubers (three independent biological replicates, respectively) were collected from *P. pedatisecta* with consistent growth for RNA sequencing. The RNA-seq data from nine samples of *P. pedatisecta* were aligned with the *P. pedatisecta* reference genome using HISAT2 with default settings. The gene expression levels were estimated using fragments per kilobase of exon per million fragments (FPKM) mapped using StringTie.

**References**

1. Shi T, Rahmani RS, Gugger PF *et al.* Distinct expression and methylation patterns for genes with different fates following a single whole-genome duplication in flowering plants. *Mol. Biol. Evol.* 2020;**37**:2394–2413.
2. Marcais G, Kingsford C. A fast, lock-free approach for efficient parallel counting of occurrences of k-mers. *Bioinformatics*, 2011;**27**:764–770.
3. Qian ZH, Li Y, Yang JS *et al.* The chromosome‐level genome of a free‐floating aquatic weed *Pistia stratiotes* provides insights into its rapid invasion. *Mol. Ecol. Resour.* 2022;**22**(7):2732–2743.
4. Belton JM, McCord RP, Gibcus JH *et al.* Hi-C: a comprehensive technique to capture the conformation of genomes. *Methods*, 2012;**58**:268–276.
5. Koren S, Walenz BP, Berlin K *et al.* Canu: scalable and accurate long-read assembly via adaptive k-mer weighting and repeat separation. *Genome Res.* 2017;**27**:722–736.
6. Walker BJ, Abeel T, Shea T *et al.* Pilon: an integrated tool for comprehensive microbial variant detection and genome assembly improvement. *Plos One* 2014;**9**:e112963.
7. Li H, Durbin R. Fast and accurate short read alignment with Burrows-Wheeler transform. *Bioinformatics* 2009;**25**:1754–1760.
8. Burton JN, Adey A, Patwardhan RP *et al.* Chromosome-scale scaffolding of de novo genome assemblies based on chromatin interactions. *Nat. Biotechnol.* 2013;**31**:1119–1125.
9. Durand NC, Shamim MS, Machol I *et al.* Juicer provides a One-Click system for analyzing loop-resolution Hi-C experiments. *Cell Syst.* 2016;**3**:95–98.
10. Seppey M, Manni M, Zdobnov EM. BUSCO: assessing genome assembly and annotation completeness. *Methods Mol. Biol.* 2019;**1962**:227–245.
11. Rhie A, Walenz BP, Koren S *et al.* Merqury: reference-free quality, completeness, and phasing assessment for genome assemblies. *Genome Biol.* 2020;**21**:245.
12. Flynn JM, Hubley R, Goubert C *et al.* RepeatModeler2 for automated genomic discovery of transposable element families. *Proc. Natl. Acad. Sci. USA* 2020;**117**:9451–9457.
13. Xu Z, Wang H. LTR_FINDER: an efficient tool for the prediction of full-length LTR retrotransposons. *Nucleic Acids Res.* 2007;**35**:W265–268.
14. Ellinghaus D, Kurtz S, Willhoeft U. LTRharvest, an efficient and flexible software for de novo detection of LTR retrotransposons. *BMC Bioinformatics* 2008;**9**:18.
15. Ou S, Jiang N. LTR_retriever: A highly accurate and sensitive program for identification of long terminal repeat retrotransposons. *Plant Physiol.* 2018;**176**:1410–1422.
16. Ma JX, Bennetzen JL. Rapid recent growth and divergence of rice nuclear genomes. *P. Natl. Acad. Sci. USA* 2004;**101**:12404–12410.
17. Bruna T, Hoff KJ, Lomsadze A *et al.* BRAKER2: automatic eukaryotic genome annotation with GeneMark-EP+ and AUGUSTUS supported by a protein database. *NAR Genom. Bioinform.* 2021;**3**:lqaa108.
18. Keilwagen J, Wenk M, Erickson JL *et al.* Using intron position conservation for homology-based gene prediction. *Nucleic Acids Res.* 2016;**44**:e89.
19. Kim D, Paggi JM, Park C *et al.* Graph-based genome alignment and genotyping with HISAT2 and HISAT-genotype. *Nat. Biotechnol.* 2019;**37**:907.
20. Pertea M, Pertea GM, Antonescu CM *et al.* StringTie enables improved reconstruction of a transcriptome from RNA-seq reads. *Nature Biotechnol.* 2015;**33**:290.
21. Grabherr MG, Haas BJ, Yassour M *et al.* Full-length transcriptome assembly from RNA-Seq data without a reference genome. *Nat. Biotechnol.* 2011;**29**:644–U130.
22. Haas BJ, Delcher AL, Mount SM *et al.* Improving the *Arabidopsis* genome annotation using maximal transcript alignment assemblies. *Nucleic Acids Res.* 2003;**31**:5654–5666.
23. Haas BJ, Salzberg SL, Zhu W *et al.* Automated eukaryotic gene structure annotation using EVidenceModeler and the program to assemble spliced alignments. *Genome Biol.* 2008;**9**:R7.
24. Ashburner M, Ball CA, Blake JA *et al.* Gene ontology: tool for the unification of biology. The Gene Ontology Consortium. *Nat. Genet.* 2000;**25**:25–29.
25. Ogata H, Goto S, Sato K *et al.* KEGG: Kyoto Encyclopedia of Genes and Genomes. *Nucleic Acids Res.* 1999;**27**:29–34.
26. Hunter S, Apweiler R, Attwood TK *et al.* InterPro: the integrative protein signature database. *Nucleic Acids Res.* 2009;**37**:D211–215.
27. Emms DM, Kelly S. OrthoFinder: solving fundamental biases in whole genome comparisons dramatically improves orthogroup inference accuracy. *Genome Biol.* 2015;**16**:157.
28. Edgar RC. MUSCLE: multiple sequence alignment with high accuracy and high throughput. *Nucleic Acids Res.* 2004;**32**:1792–1797.
29. Suyama M, Torrents D, Bork P. PAL2NAL: robust conversion of protein sequence alignments into the corresponding codon alignments. *Nucleic Acids Res.* 2006;**34**:W609-W612.
30. Castresana J. Selection of conserved blocks from multiple alignments for their use in phylogenetic analysis. *Mol. Biol. Evol.* 2000;**17**:540–552.
31. Nguyen LT, Schmidt HA, von Haeseler A *et al.* IQ-TREE: A Fast and Effective Stochastic Algorithm for Estimating Maximum-Likelihood Phylogenies. *Mol. Biol. Evol.* 2015;**32**:268–274.
32. Li HT, Yi TS, Gao LM *et al.* Origin of angiosperms and the puzzle of the Jurassic gap. *Nat. Plants* 2019;**5**:461–470.
33. Mendes FK, Vanderpool D, Fulton B *et al.* CAFE 5 models variation in evolutionary rates among gene families. *Bioinformatics* 2020;**36**:5516–5518.
34. Wang YP, Tang HB, DeBarry JD *et al.* MCScanX: a toolkit for detection and evolutionary analysis of gene synteny and collinearity. *Nucleic Acids Res.* 2012;**40**:e49.
35. Yang ZH. PAML 4: Phylogenetic analysis by maximum likelihood. *Mol. Biol. Evol.* 2007;**24**:1586–1591.
36. Wang J, Sun P, Li Y *et al.* An overlooked Paleotetraploidization in Cucurbitaceae. *Mol Biol Evol.* 2018;**35**:16–26.
37. Sun PC, Jiao BB, Yang YZ *et al.* WGDI: a user-friendly toolkit for evolutionary analyses of whole-genome duplications and ancestral karyotypes. *BioRxiv* 2021;2021.04.29.441969.
38. Eddy SR. Accelerated profile HMM searches. *PLoS Comput. Biol.* 2011; **7**(10):e1002195.
39. Jones P, Binns D, Chang HY *et al.* InterProScan 5: genome-scale protein function classification. *Bioinformatics*. 2014;**30**(9):1236–1240.
40. Wang D, Zhang Y, Zhang Z *et al.* KaKs_Calculator 2.0: a toolkit incorporating gamma-series methods and sliding window strategies. *Genom. Proteom. Bioinf.* 2010;**8**(1):77–80.
41. Kumar S, Stecher G, Tamura K. MEGA7: Molecular evolutionary genetics analysis version 7.0 for big datasets. *Mol. Biol. Evol.* 2016;**33**:1870–1874.
42. Chen C, Chen H, Zhang Y *et al.*, TBtools: an integrative toolkit developed for interactive analyses of big biological data. *Mol. Plant.* 2020;**13**:1194–1202.

**2. Supplementary Figures**


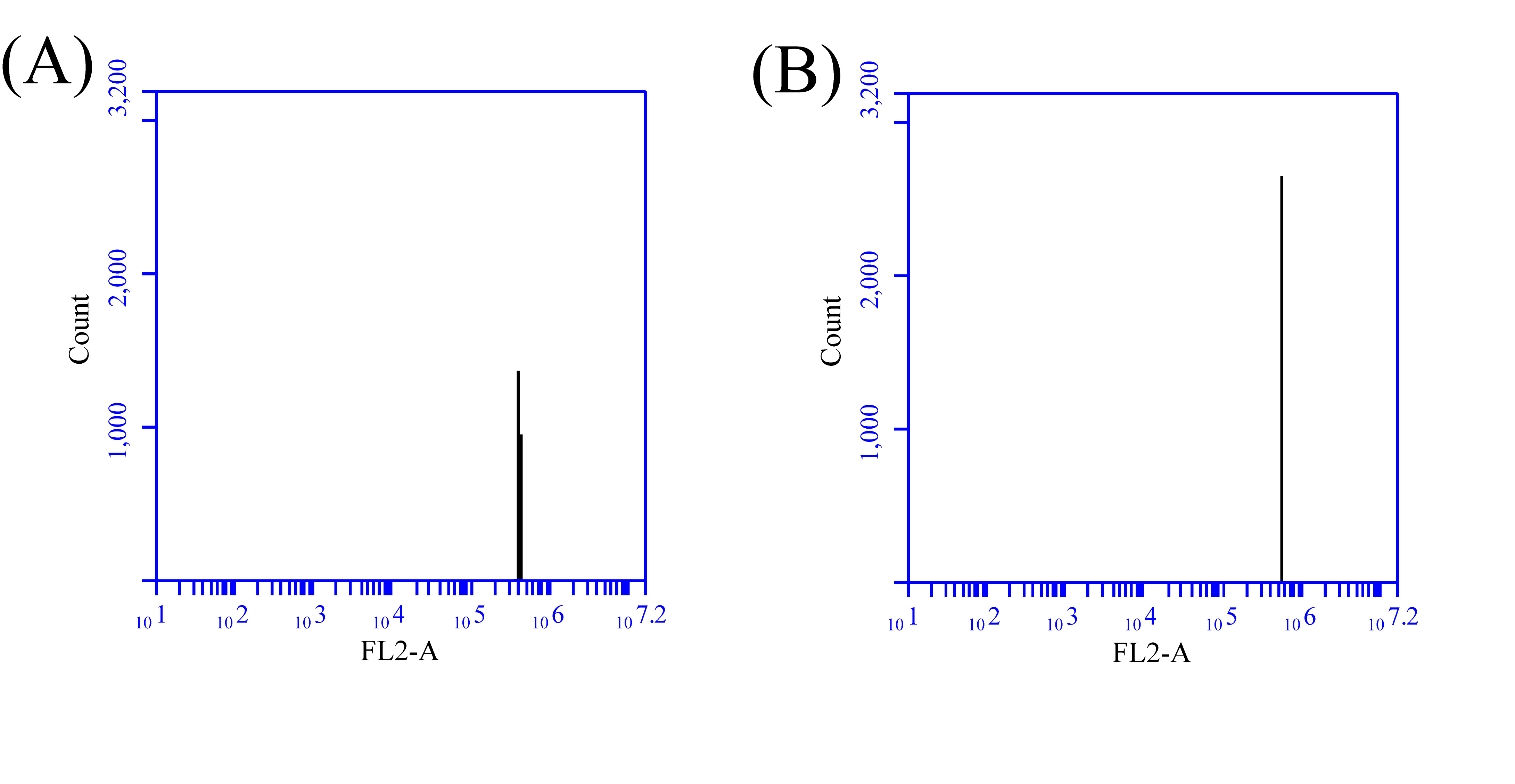


**Figure S1. Results of flow cytometry.**

(A) The genome size of *Nelumbo nucifera* was estimated 807.6Mb previously, and the fluorescence was 1,155,354. (B) *Pinellia pedatisecta*, which fluorescence was 1,483,495, and estimated the genome size to be 1036 Mb.


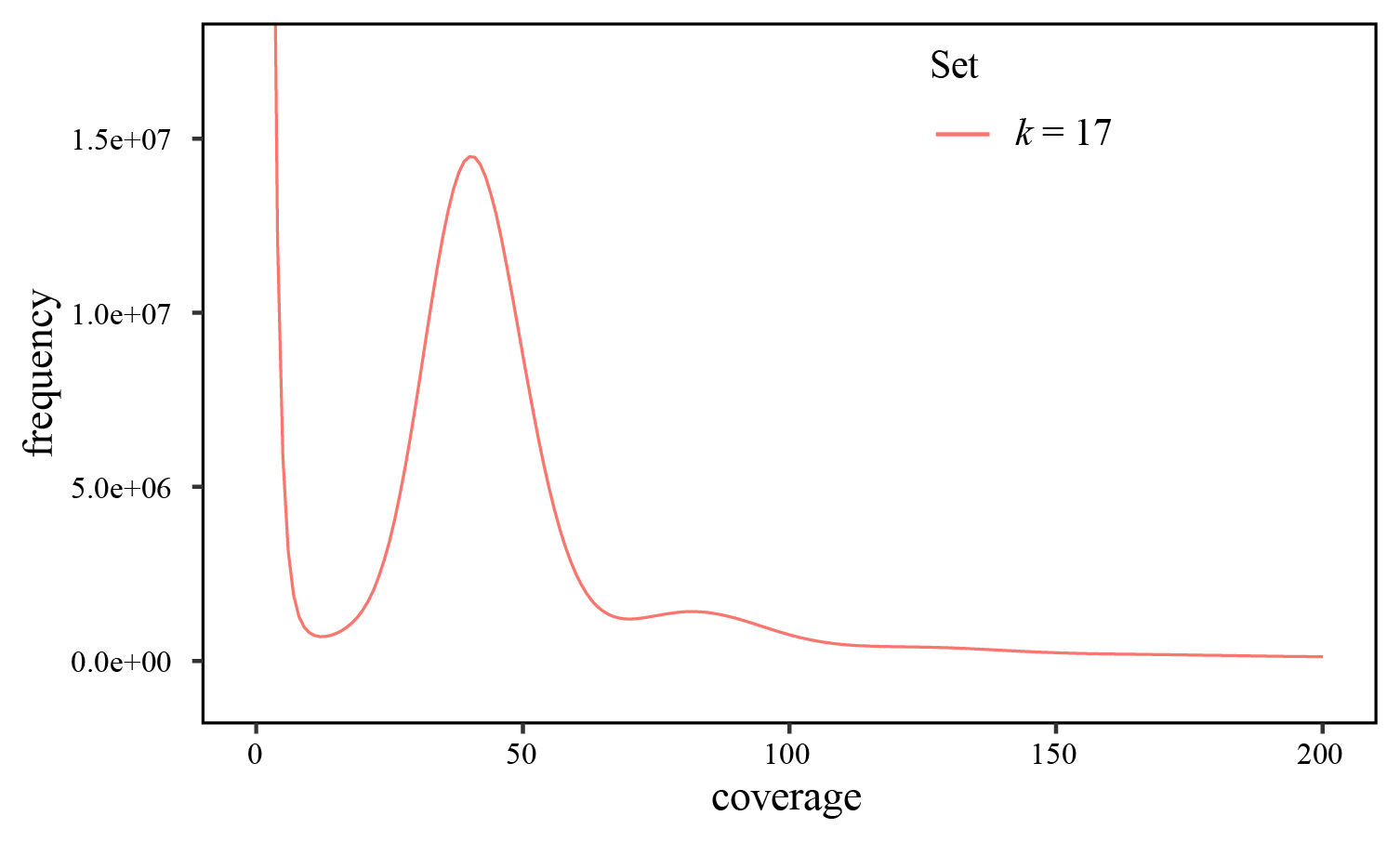


**Figure S2.** **Estimation of *P. pedatisecta* genome size based on *k-mer*** **(*k* = 17) analysis.**


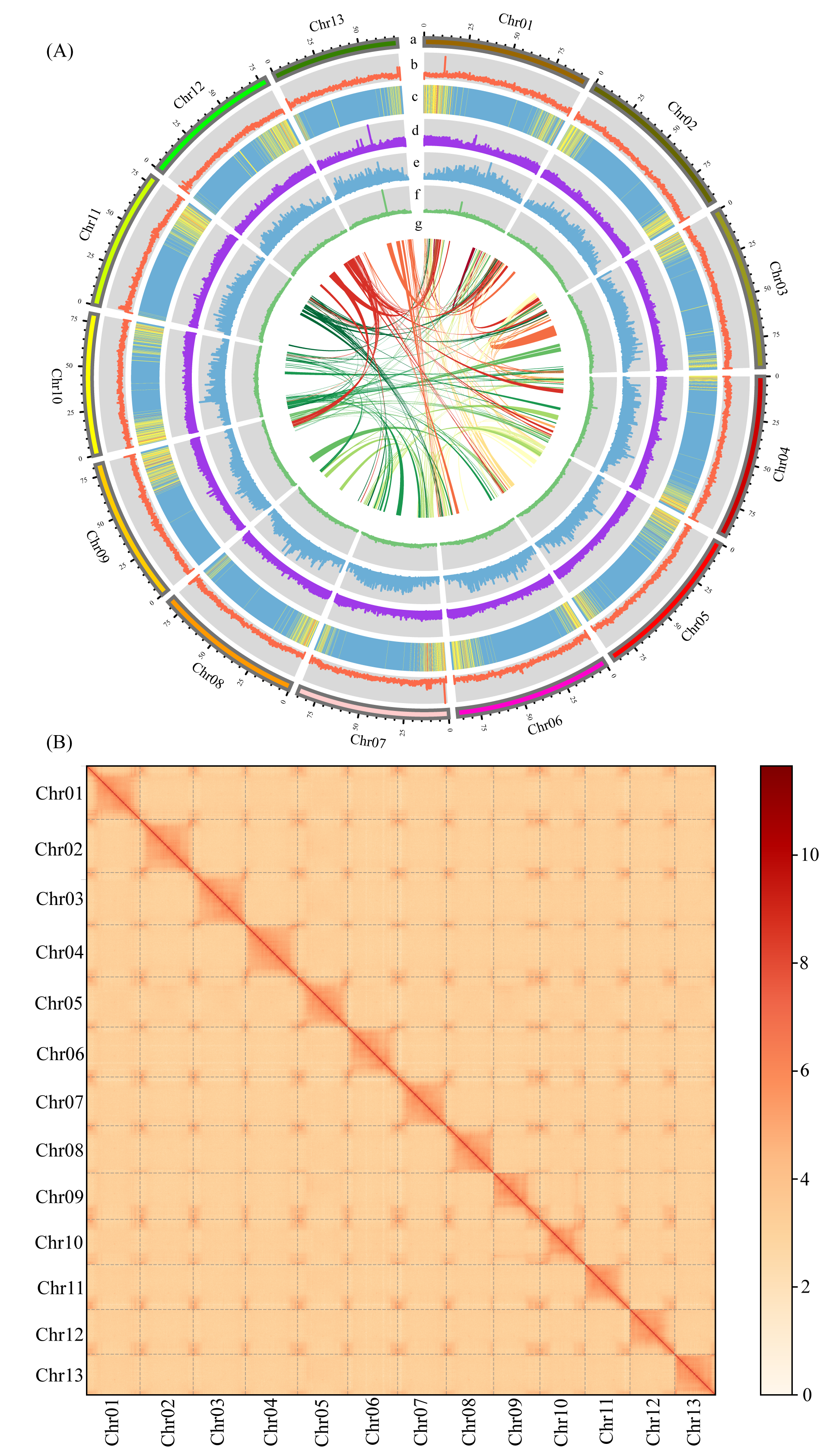


**Figure S3. Characteristics of *P. pedatisecta* genome.**

(A) Circos plot of *P. pedatisecta* genome. a, chromosome length; b, GC content; c, gene

density; d, repeat coverage; e, LTR_Gypsy density; f, LTR_Copia density; g, Syntenic blocks. (B) Hi-C interactions among the 13 chromosomes of the *P. pedatisecta* genome.


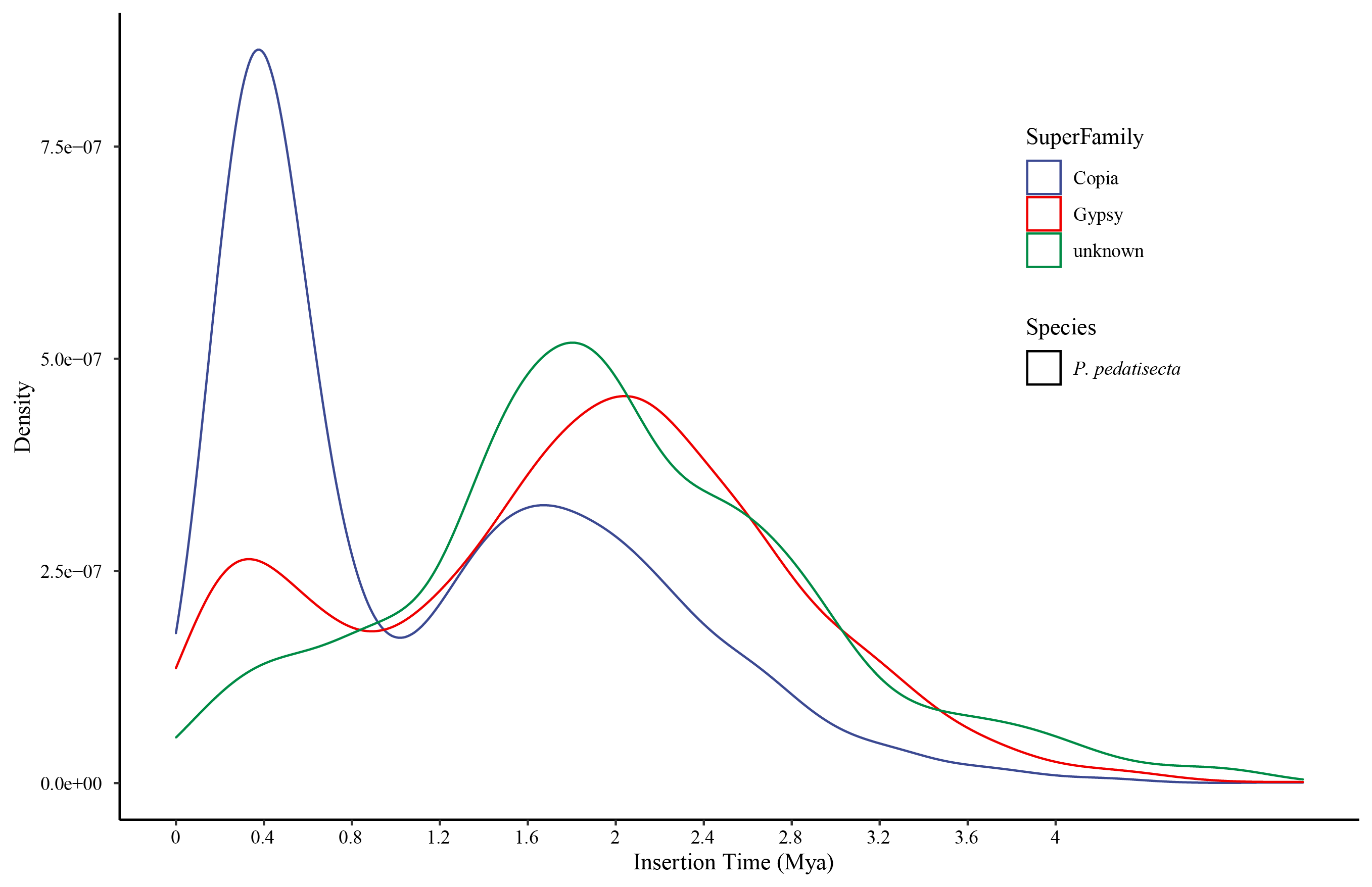


**Figure S4. The insertion time density distribution of long terminal transposon (LTR-RT) elements.**


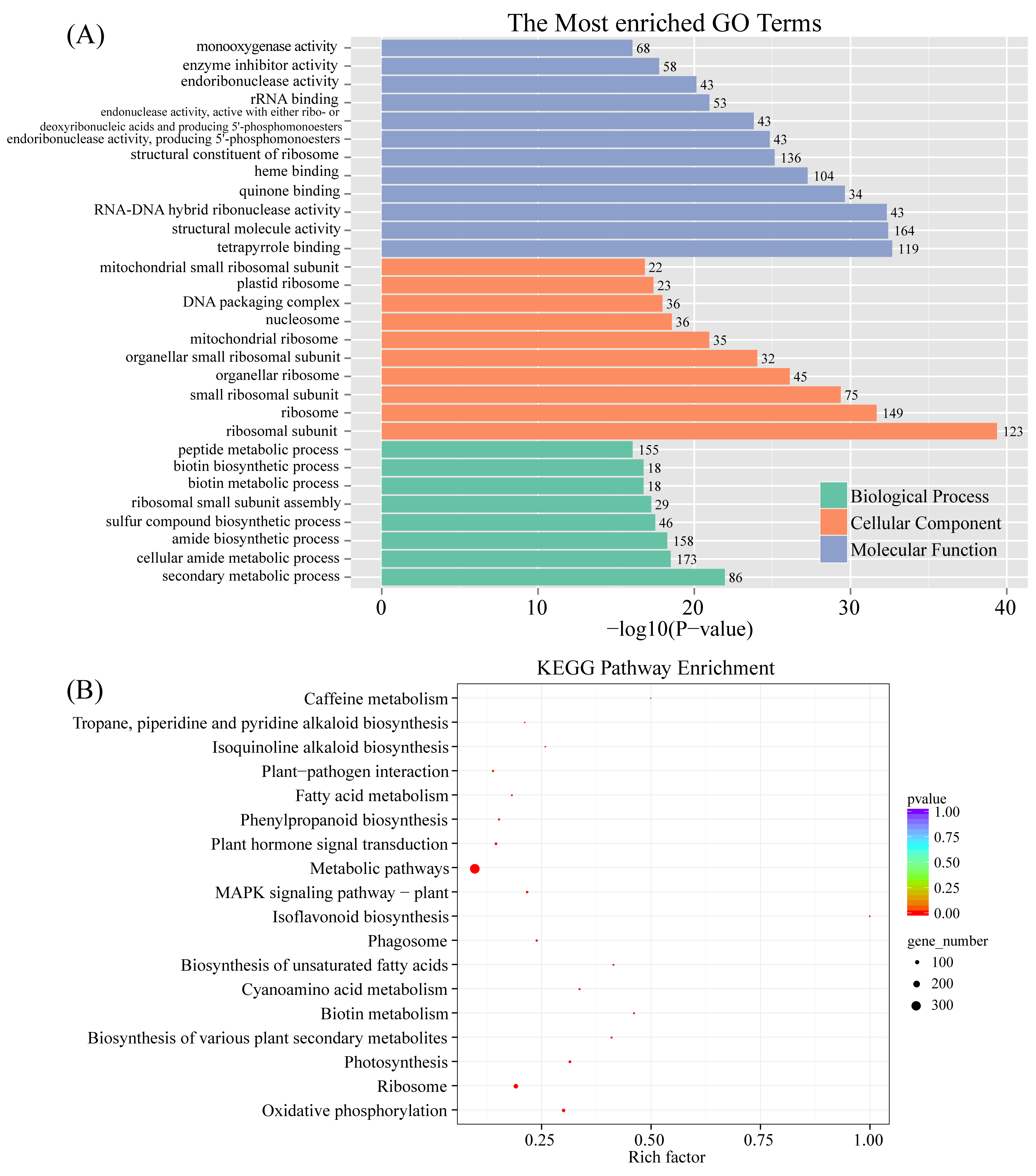


**Figure S5. Enrichment analysis of the expansion gene family.**

(A) The most enriched GO terms; (B) The analysis of KEGG enrichment.


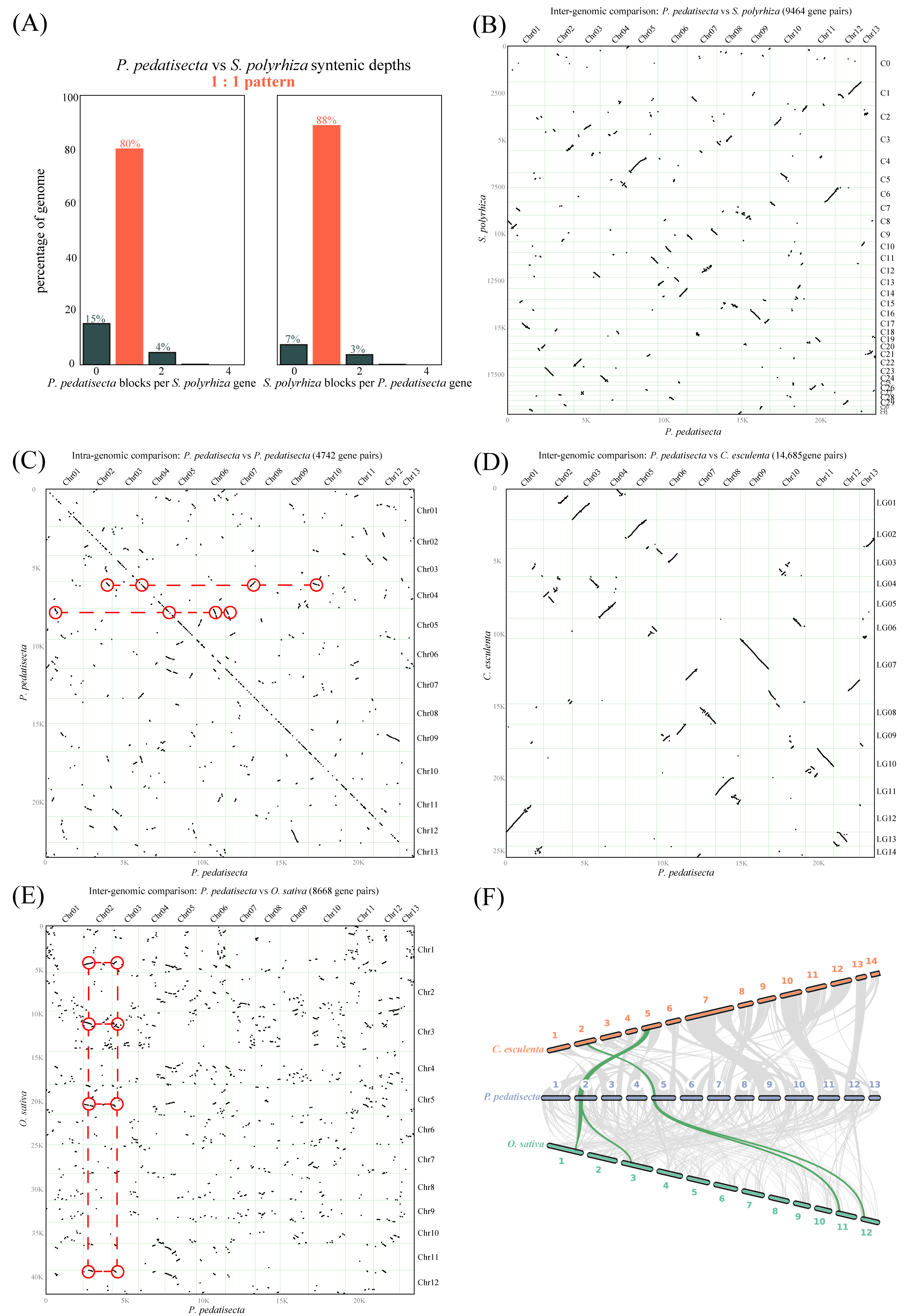


**Figure S6. Evidence for whole-genome duplication events in *P. pedatisecta*.**

(A) The syntenic depths between *P. pedatisecta* and *S. polyrhiza*; (B) The syntenic dotplot between *P. pedatisecta* and *S. polyrhiza*; (C) The syntenic dotplot of intra-*P. pedatisecta*. The red circles highlight examples of 1:4 chromosomal relationships; (D) The syntenic dotplot between *P. pedatisecta* and *C. esculenta*; (E) The syntenic dotplot between *P. pedatisecta* and *O.sativa*. The red circles highlight an example of 2:4 chromosomal relationships. (F) The karyotype figure of *P. pedatisecta*, *O. sativa* and *C. esculenta*. The green line shows an example of two syntenic blocks.


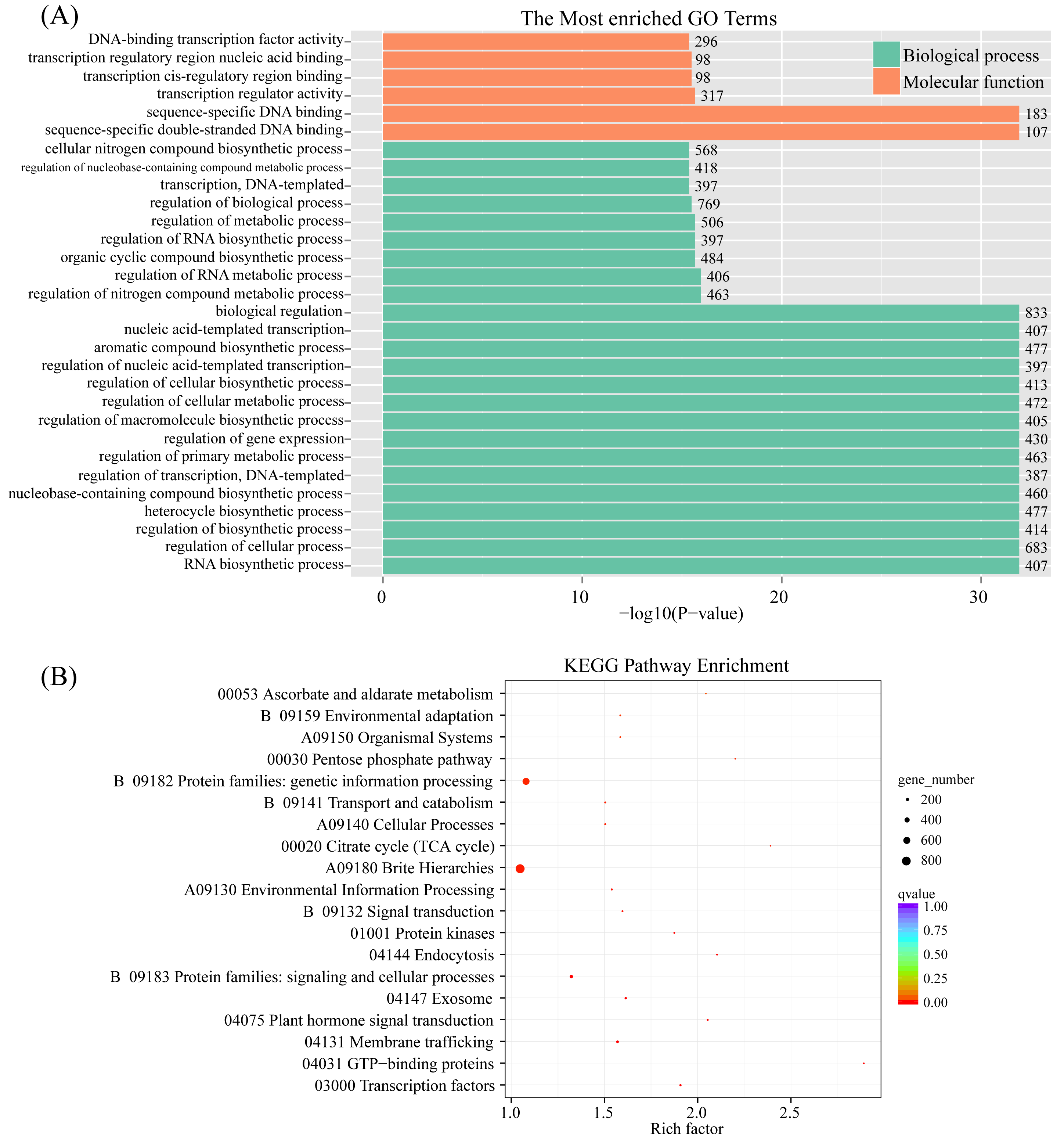


**Figure S7. Enrichment analysis of** **the duplicated genes after WGDs.**

(A) The most enriched GO terms; (B) The analysis of KEGG enrichment.


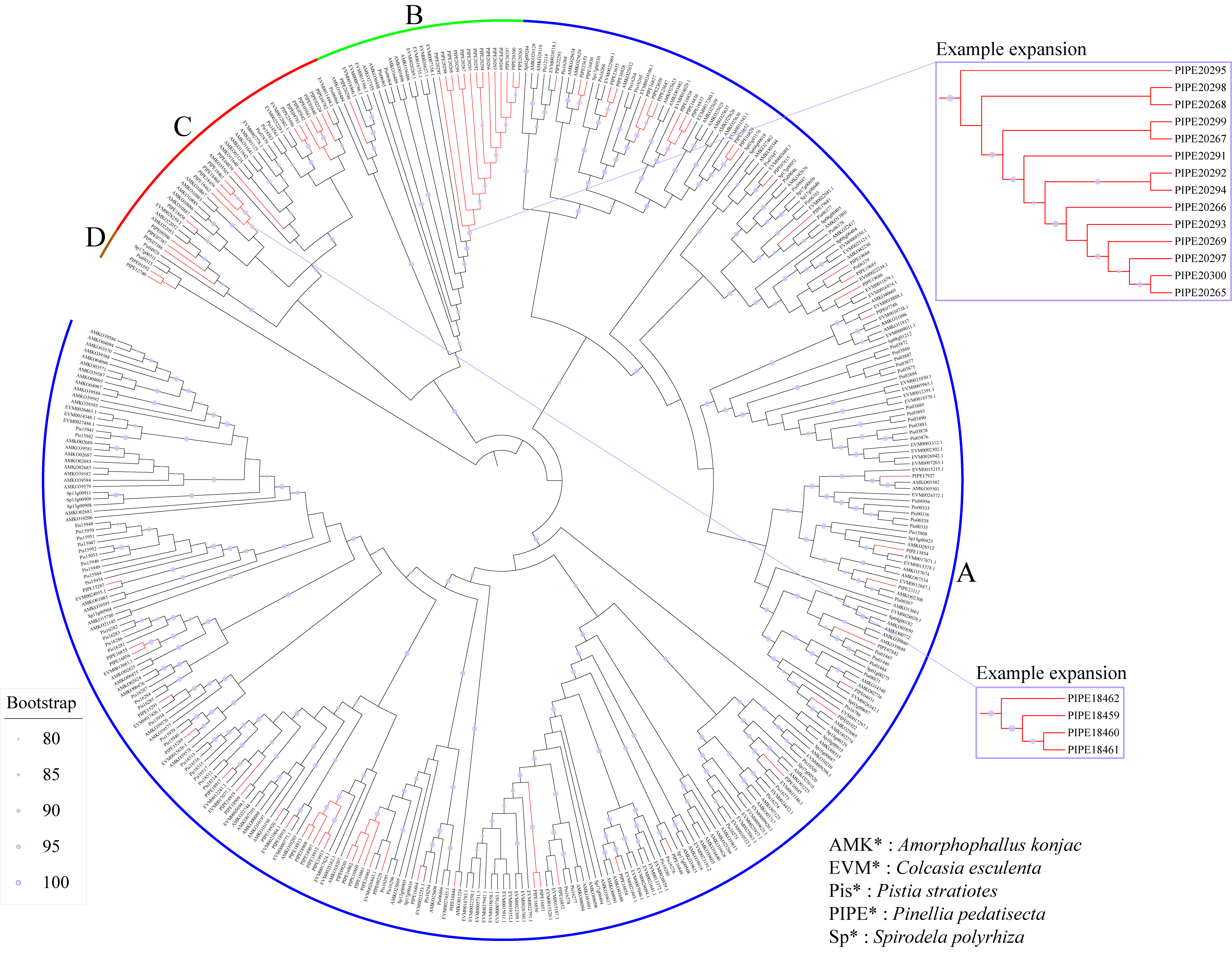


**Figure S8. The phylogenetic tree of *Galanthus nivalis* agglutinin genes from five species.** The dots size indicates bootstraps >80; red branches denote PPA genes. Sub-tree within purple box is the example of PPA genes lineage-specific expansion present in *P. pedatisecta*.


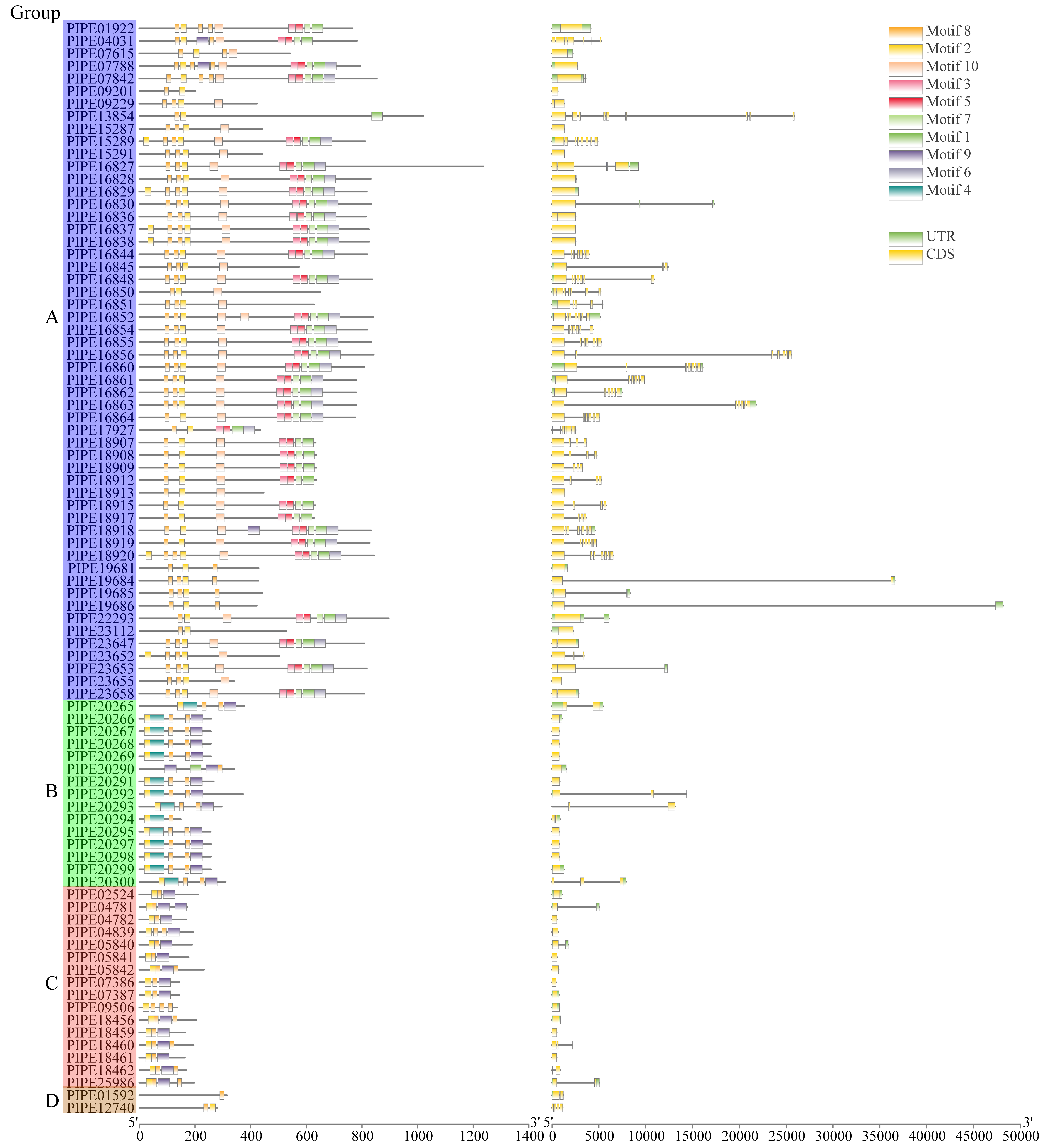


**Figure S9. The characteristics of conserved motifs and gene structures in PPA genes.**

**3. Supplementary Tables**

**Table S1. Classification of repetitive elements in the *P. pedatisecta***

| Repeat types | | Number of elements | Repeat size (bp) | Percentage of the genome (%) |
| --- | --- | --- | --- | --- |
| DNA |  | 30,386 | 25,019,885 | 2.12 |
| LINEs |  | 46,905 | 35,514,461 | 3.00 |
| LTR |  | 214,650 | 463,913,327 | 39.23 |
|  | Pao | 255 | 54,247 | 0 |
|  | Copia | 74,556 | 171,851,298 | 14.53 |
|  | Gypsy | 139,582 | 291,712,575 | 24.67 |
| Unclassified |  | 851,908 | 374,039,149 | 31.63 |
| Simple repeats |  | 209,279 | 18,277,426 | 1.55 |
| Low complexity |  | 19,477 | 1,061,550 | 0.09 |
| Total interspersed repeats |  |  | 898,486,822 | 75.98 |
| Total |  |  | 918,368,989 | 77.66 |

**Table S2. Statistics of gene annotation using different databases**

| Database | Annotated number | Percentage (%) |
| --- | --- | --- |
| Swiss-Prot | 16,480 | 63.11 |
| InterPro | 21,629 | 82.83 |
| KOG | 7133 | 27.32 |
| GO | 18,572 | 71.12 |
| KEGG | 8226 | 31.5 |
| Nr | 22,748 | 87.11 |
| Annotated total | 24,126 | 92.39 |
| Gene total | 26,113 | 100 |

**Table S3. Summary of BUSCOs analysis of *P. pedatisecta* genome**

|  | Genome | | Gene sets | | |
| --- | --- | --- | --- | --- | --- |
|  | Count | Percentage (%) | | Count | Percentage (%) |
| Complete BUSCOs | 1474 | 91.3 | | 1554 | 96.2 |
| Complete and single-copy BUSCOs | 1396 | 86.5 | | 1455 | 90.1 |
| Complete and duplicated BUSCOs | 78 | 4.8 | | 99 | 6.1 |
| Fragmented BUSCOs | 51 | 3.2 | | 30 | 1.9 |
| Missing BUSCOs | 89 | 5.5 | | 30 | 1.9 |
| Total BUSCO groups searched | 1614 | 100.0 | | 1614 | 100 |

**Table S4. Pairwise comparison of *Ka* and *Ks* values for each PPA gene pair in *P. pedatisecta***

| Syntenic gene pairs | *Ka* | *Ks* | *Ka/Ks* | Duplication type |
| --- | --- | --- | --- | --- |
| PIPE04781 & PIPE04782 | 0.16 | 0.31 | 0.52 | TD |
| PIPE04839 & PIPE05840 | 0.47 | 0.65 | 0.73 | TD |
| PIPE05840 & PIPE05841 | 0.09 | 0.11 | 0.82 | TD |
| PIPE05841 & PIPE05842 | 0.29 | 0.31 | 0.94 | TD |
| PIPE07386 & PIPE07387 | 0.01 | 0.03 | 0.36 | TD |
| PIPE16827 & PIPE16828 | 0.27 | 0.73 | 0.37 | TD |
| PIPE16828 & PIPE16829 | 0.39 | 1.54 | 0.26 | TD |
| PIPE16829 & PIPE16830 | 0.44 | 1.68 | 0.26 | TD |
| PIPE16836 & PIPE16837 | 0.02 | 0.07 | 0.36 | TD |
| PIPE16837 & PIPE16838 | 0.02 | 0.04 | 0.47 | TD |
| PIPE16844 & PIPE16845 | 0.60 | 1.28 | 0.47 | TD |
| PIPE16850 & PIPE16851 | 0.07 | 0.17 | 0.42 | TD |
| PIPE16851 & PIPE16852 | 0.27 | 0.83 | 0.32 | TD |
| PIPE16854 & PIPE16855 | 0.52 | 2.4 | 0.21 | TD |
| PIPE16855 & PIPE16856 | 0.05 | 0.2 | 0.24 | TD |
| PIPE16860 & PIPE16861 | 0.19 | 0.47 | 0.40 | TD |
| PIPE16861 & PIPE16862 | 0.07 | 0.18 | 0.37 | TD |
| PIPE16862 & PIPE16863 | 0.11 | 0.26 | 0.41 | TD |
| PIPE16863 & PIPE16864 | 0.35 | 1.15 | 0.31 | TD |
| PIPE18459 & PIPE18460 | 0.12 | 0.3 | 0.39 | TD |
| PIPE18460 & PIPE18461 | 0.11 | 0.3 | 0.37 | TD |
| PIPE18461 & PIPE18462 | 0.15 | 0.17 | 0.88 | TD |
| PIPE18907 & PIPE18908 | 0.06 | 0.24 | 0.26 | TD |
| PIPE18908 & PIPE18909 | 0.22 | 0.64 | 0.34 | TD |
| PIPE18912 & PIPE18913 | 0.12 | 0.32 | 0.39 | TD |
| PIPE18917 & PIPE18918 | 0.16 | 0.42 | 0.38 | TD |
| PIPE18918 & PIPE18919 | 0.12 | 0.42 | 0.30 | TD |
| PIPE18919 & PIPE18920 | 0.12 | 0.36 | 0.33 | TD |
| PIPE19684 & PIPE19685 | 0.17 | 0.31 | 0.55 | TD |
| PIPE19685 & PIPE19686 | 0.19 | 0.5 | 0.39 | TD |
| PIPE20265 & PIPE20266 | 0.06 | 0.16 | 0.40 | TD |
| PIPE20266 & PIPE20267 | 0.05 | 0.14 | 0.36 | TD |
| PIPE20267 & PIPE20268 | 0.06 | 0.23 | 0.26 | TD |
| PIPE20268 & PIPE20269 | 0.08 | 0.24 | 0.32 | TD |
| PIPE20290 & PIPE20291 | 0.59 | 0.81 | 0.73 | TD |
| PIPE20291 & PIPE20292 | 0.12 | 0.27 | 0.44 | TD |
| PIPE20292 & PIPE20293 | 0.10 | 0.2 | 0.49 | TD |
| PIPE20293 & PIPE20294 | 0.15 | 0.42 | 0.36 | TD |
| PIPE20294 & PIPE20295 | 0.11 | 0.26 | 0.41 | TD |
| PIPE20297 & PIPE20298 | 0.08 | 0.25 | 0.32 | TD |
| PIPE20298 & PIPE20299 | 0.06 | 0.22 | 0.27 | TD |
| PIPE20299 & PIPE20300 | 0.08 | 0.2 | 0.41 | TD |
| PIPE23652 & PIPE23653 | 0.58 | 1.74 | 0.34 | TD |
| PIPE02524 & PIPE04839 | 0.40 | 0.55 | 0.72 | WGD |
| PIPE20265 & PIPE20297 | 0.07 | 0.16 | 0.48 | WGD |
| PIPE20266 & PIPE20292 | 0.07 | 0.15 | 0.44 | WGD |
| PIPE20267 & PIPE20291 | 0.07 | 0.19 | 0.37 | WGD |
| PIPE20268 & PIPE20290 | 0.60 | 0.95 | 0.63 | WGD |

Note: *Ka*, the non-synonymous substitution rate; *Ks*, the synonymous substitution rate; TD, tandem duplication; WGD, whole-genome duplication.

**Table S5. Statistics of characteristics of *P. pedatisecta* genome by *k-mer* (*k* = 17) analysis**

| *k-mer* | *k-mer* number | *k-mer* coverage | Genome size (Mb) | Heterozygous ratio (%) | Repeat (%) |
| --- | --- | --- | --- | --- | --- |
| 17 | 48,598,762,005 | 40 | 1177 | 0.25 | 62.48 |

| **Table S6. Sequencing and quality filtering statistics** | | | | |
| --- | --- | --- | --- | --- |
| Type | Total base (Gb) | Reads number | Sequence coverage (×) | Read length |
| Illumina | 108.81 | 362,691,649 | 92.03 | 150bp |
| Pacbio | 136.23 | 7,621,820 | 115.22 | 28,489bp (N50) |
| HIC | 116.01 | 437,728,812 | 98.12 | 150bp |
| Total | 361.05 | 808,042,281 | 305.36 |  |

**Table S7. Download links for genomic profiles used in this study**

| Species | Database | Url |
| --- | --- | --- |
| *Arabidopsis thaliana* | Pyhtozome | https://phytozome-next.jgi.doe.gov/info/Athaliana_TAIR10 |
| *Amborella trichopoda* | Pyhtozome | https://phytozome-next.jgi.doe.gov/info/Atrichopoda_v1_0 |
| *Solanum lycopersicum* | Pyhtozome | https://phytozome-next.jgi.doe.gov/info/Slycopersicum_ITAG4_0 |
| *Vitis vinifera* | Pyhtozome | https://phytozome-next.jgi.doe.gov/info/Vvinifera_v2_1 |
| *Nelumbo nucifera* | - | http://nelumbo.biocloud.net/nelumbo/home |
| *Aquilegia coerulea* | Pyhtozome | https://phytozome-next.jgi.doe.gov/info/Acoerulea_v3_1 |
| *Liriodendron chinense* | Hardwood Genomics Project | https://www.hardwoodgenomics.org/Genome-assembly/2630420?tripal_pane=group_downloads |
| *Ananas comosus* | Pyhtozome | https://phytozome-next.jgi.doe.gov/info/Acomosus_v3 |
| *Colcasia esculenta* | CNGBdb | https://db.cngb.org/search/project/CNP0001082/ |
| *Oryza sativa* | Pyhtozome | https://phytozome-next.jgi.doe.gov/info/Osativa_v7_0 |
| *Musa acuminata* | Pyhtozome | https://phytozome-next.jgi.doe.gov/info/Macuminata_v1 |
| *Zostera marina* | JGI | https://data.jgi.doe.gov/refine-download/phytozome?organism=Zmarina&expanded=Phytozome-668 |
| *Spirodela polyrhiza* | Pyhtozome | https://phytozome-next.jgi.doe.gov/info/Spolyrhiza_v2 |
| *Solanum tuberosum* | Pyhtozome | https://data.jgi.doe.gov/refine-download/phytozome?genome_id=686 |
| *Pistia stratiotes* | CNGBdb | https://db.cngb.org/search/assembly/CNA0036302/ |
| *Amorphophallus konjac* | - | https://doi.org/10.6084/m9.figshare.15169578 |
| *Lemna minuta* | - | https://genomevolution.org/coge/SearchResults.pl?s=61245&p=genome |

**Table S8. Downloaded sequences from GenBank in this study**

| Species | Accession No. | Renaming the sequence |
| --- | --- | --- |
| *Pinellia pedatisecta* | AAR27793.1 | PPA1 |
|  | AGV40779.1 | PPA2 |
|  | ADK56179.1 | PPA3 |
| *Pinellia ternata* | ADD91328.1 | PTA1 |
|  | QNL35375.1 | PTA2 |
|  | AFY06641.1 | PTA3 |
|  | AAR27794.1 | PTA4 |
|  | AGV40777.1 | PTA5 |
|  | AAU29612.1 | PTA6 |
|  | ABX47148.1 | PTA7 |
|  | AAP20876.1 | PTA8 |
|  | AAZ05446.1 | PTA9 |
|  | AEZ35184.1 | PTA10 |
| *Pinellia cordata* | ABK88277.1 | PCA1 |
|  | AGV40778.1 | PCA2 |
| *Pinellia integrifolia* | AOA49622.1 | PIA |
